# Emotive Themes from Tennessee Cattle Producers Regarding Responsible Antibiotic Use

**DOI:** 10.3390/ani12162088

**Published:** 2022-08-16

**Authors:** Chika C. Okafor, John E. Ekakoro, Marc Caldwell, Elizabeth B. Strand

**Affiliations:** 1 Department of Biomedical and Diagnostic Sciences, College of Veterinary Medicine, University of Tennessee, Knoxville, TN 37996, USA; 2 Department of Large Animal Clinical Sciences, College of Veterinary Medicine, University of Tennessee, Knoxville, TN 37996, USA

**Keywords:** antimicrobial resistance, antimicrobial use, cattle

## Abstract

**Simple Summary:**

Understanding the emotional experiences of producers towards their antimicrobial use (AMU) practices can be a starting point to making future behavioral changes that could reduce the emergence of antimicrobial resistance challenge. Between June 2017 and March 2018, seven focus group meetings of Tennessee (TN) beef and dairy cattle producers were conducted to evaluate producers’ emotional views regarding responsible AMU in cattle. Sixty-two TN cattle producers participated and emotively expressed the following: (1) deep connections to animals in ways that improve animal and public health; (2) pride in their quality of products; (3) distress that consumers misconceive their AMU practices as indiscriminate; and (4) recommended that producers be more transparent about their AMU practices and the public improve their awareness for detecting marketers’ deceptive product labels that take advantage of public ignorance. Knowledge of these producers’ emotions would help educators target more successful behavioral change campaigns, improving stewardship in AMU practices among producers.

**Abstract:**

To improve judicious antimicrobial use (AMU) in food animals in the United States, all feed additives that were medically important antimicrobials were moved from over the counter to Veterinary Feed Directive in 2017. This action required a change in behavior of producers’ AMU practices. Because emotions are important aspects of behavior, several behavioral interventions have targeted people’s emotions as means of effecting change. Hence, understanding and incorporating the emotional experiences of producers towards current AMU practices can be a starting point to making future behavioral changes that could reduce the emergence of antimicrobial resistance challenge. Between June 2017 and March 2018, seven focus group meetings of Tennessee (TN) beef and dairy cattle producers were conducted to evaluate producers’ emotional views regarding responsible AMU in TN cattle. Sixty-two TN cattle producers participated and emotively expressed the following: (1) deep connections to animals in ways that improve animal and public health; (2) pride in their quality of products; (3) distress that consumers misconceive their AMU practices as indiscriminate; and (4) recommended that producers be more transparent about their AMU practices and the public improve their awareness for detecting marketers’ deceptive product labels that take advantage of public ignorance. Knowledge of these producers’ emotions would help educators target more successful behavioral change campaigns, improving stewardship in AMU practices among producers.

## 1. Introduction

To prevent potential public health consequences of antimicrobial resistance (AMR), many countries have instituted measures to reduce and minimize antimicrobial use (AMU) in food animals [1,2]. All AMU practices (judicious and non-judicious) in animals for both therapeutic and non-therapeutic purposes may propagate the shedding of substantial amounts of AMR microorganisms [2,3,4]. Currently available evidence on the impact of AMU in food animals on AMR in human pathogens is debatable [5,6,7,8,9,10,11,12,13]. Despite the controversies around the impacts of AMU in animals on public health, judicious practices among producers are widely used by all sectors within the animal agriculture food production system to prolong the efficacy of current antimicrobial agents [14,15,16].

Adoption of preventive measures in the face of uncertainty and exploration of various alternatives to potential threats is a guiding tenet of public health [17]. To preserve the efficacy of medically important antimicrobials, the World Health Organization (WHO, Geneva, Switzerland) recommended complete restriction of AMU for growth promotion and disease prevention in food-producing animals [18]. Consequently, in the US, the Food and Drug Administration (FDA, Silver Spring, MD, USA) completed the implementation of Guidance For Industry #213, effective from 1 January 2017, authorizing that medically important antimicrobials may only be used in feed or drinking water of food producing animals with veterinary oversight (i.e., when authorized by a prescription, or for feed drugs, by a Veterinary Feed Directive (VFD)) and can no longer be used for production (e.g., growth promotion) purposes [19]. So, success of judicious AMU interventions, such as the implementation of Guidance For Industry #213, would require a change in previous AMU practices, particularly among cattle producers. This change would not come easy because producers consistently base their production actions and decisions on a complex system of core values that affect their farms, families, and businesses [20].

To facilitate prudent AMU in animal production, an emphasis on the agricultural education of cattle producers on prudent AMU practices is critical [21]. Generic educational campaigns may not be so effective in this course, because embarking on an intensive effort to change behavior among many people requires an understanding of the theory of planned behavior [22]. Specifically, this approach has been recommended in the wide-spread efforts to identify and change AMU beliefs by those who have considerable influence over AMU in animal agriculture [23]. More recently, psychological and behavioral determinants of farmers and veterinarians concerning AMU in food animal agriculture have been studied [24,25,26,27,28], because understanding these values can be a starting point to shaping future behavioral changes that could reduce the emergence of AMR challenge.

Because emotions are important aspects of behavior [29], several behavioral interventions have targeted people’s emotions as means of effecting change [30,31,32]. Therefore, the objective of this study was to evaluate producers’ emotional views regarding responsible AMU in cattle in Tennessee (TN). These findings could influence targeted campaigns to adopt statewide and nationwide stewardship of AMU, leading to faster behavioral changes that would improve judicious AMU practices. In the absence of this knowledge, generic educational campaigns to these cattle producers on judicious AMU could be non-productive.

## 2. Materials and Methods

### 2.1. Research Team and Reflexivity

The research team comprised of a female (E.B.S.) and 3 males (C.C.O., J.E.E., and M.C.) who were all employees of the University of Tennessee, Knoxville, at the time of the study. All the focus group discussions were moderated by one individual (E.B.S.), a veterinary social work specialist with expertise in conducting focus group interviews and in the behavioral sciences. The moderator guided the focus groups to allow development of free discussion throughout the topics while the others (C.C.O., J.E.E., and M.C.) took handwritten field notes of key points, provided clarifications to questions, and asked follow-up questions when necessary. The first author (C.C.O.) is experienced in the design, conduct, and analysis of qualitative epidemiological research. The second author (J.E.E.) is trained in qualitative research methods and analysis. The third author (M.C.) is a livestock veterinary specialist and actively engaged in food animal veterinary practice in TN. Each member of the research team has a doctoral degree in their respective areas of expertise (J.E.E. was in doctoral training at the time of the study).

### 2.2. Study Design

No relationship was established between the research team and the cattle producers (participants) prior to study commencement; however, the participants were not randomly selected. Between June 2017 and March 2018, the leadership of the Tennessee Cattlemen’s Association (TCA, Murfreesboro, TN, USA) purposively invited 12 members (either through face-to-face, telephone, or email) with experience in different cattle production systems for each focus group. Prior to recruitment, the TCA leadership introduced the research team and the objectives of the study to the participants. Hence, participants knew the researchers’ institutional affiliation and reasons for conducting the research in advance. However, the research team disclosed their unbiased position on the topic of antibiotic use in animal production at the beginning of the focus group discussions. Focus group sites were selected to accommodate all regions (East, Middle, and West) of TN as well as to be representative of the demographic density of the type of cattle operations (beef or dairy) in the state. The focus group meetings were held in either restaurants or county extension centers using a semi-structured interview guide. Participants could opt out of the focus groups at any time. All invited participants were provided with a meal irrespective of their active participation. All participants read and signed a consent to participate in the study and the meetings were held exclusively between the researchers and the participants. For anonymity and confidentiality, participants were assigned numerical identities. Each participant announced their identifier before speaking and referenced fellow participants by their identity numbers. A semi-structured interview guide which was modified after the first focus group was utilized (see additional files 1 and 2). The modified interview guide (additional file 2) consisted of 11 open-ended questions. Each question was boldly displayed on a large post-it board to the visibility of the participants. Following each focus group meeting and before the next one, the research team held a debriefing session to discuss emerging themes and compare field notes between focus groups for an evaluation of data saturation. Each focus group discussion lasted approximately 90 min and was captured on video and audio recordings.

### 2.3. Analysis

Afterwards, captured data were rendered and transcribed by a licensed commercial transcription service provider. No further contacts were made with the participants and no corrections were made after the transcription. Transcripts were imported into a commercial qualitative data analysis software (NVivo; QSR International Pty Ltd. Version 11, Doncaster, Australia, 2017) for further coding and thematic analysis (TA). A six-phase process (data familiarization and writing familiarization notes; systematic data coding; generating initial themes from coded and collected data; developing and reviewing themes; refining, defining, and naming themes; and writing the report) for data management, coding and theme development was utilized [33]. A reflexive TA approach which was more deductive was utilized for the analysis [33].

Briefly, all four researchers wrote familiarization notes immediately after each focus group, individually read the transcript and created independent nodes of themes. Two team members (C.C.O. and E.B.S.) searched for, reviewed, and identified themes that met the objective of the present study. Collectively, the entire team re-evaluated the identified themes and corresponding excerpts and shared suggestions. Afterwards, the two team members (C.C.O. and E.B.S.) refined these themes to produce major and minor-themes and ensured that each major theme was meaningful and clear but distinct from other themes [34]. Participants’ quotations were presented to illustrate the themes and each quotation was identified by participant number and location of focus group, except for a few un-identified participants. Finally, a thematic map was used to display the identified themes.

## 3. Results

Five beef and two dairy focus groups were conducted and 62 Tennessee cattle producers, comprising 58 males and 4 females, participated in this study (additional file 3). Participants’ perceived ages ranged from late 20s to early 70s and the reported herd size per producer ranged from approximately 20 to 1100 cattle.

Following the debriefing session at the end of the fifth beef producers’ focus group, substantial new themes were not captured in comparison to the themes identified in the preceding four focus groups. Hence, an interpretative judgement of data saturation was made at the fifth beef producers’ focus group but could not be evaluated in the dairy producers’ focus groups. Given that dairy cattle production is not predominant in TN, with about 40,000 cattle in less than 1000 farms [35], no further focus group was sought after the second dairy producers’ focus group. Albeit, both focus groups yielded similar themes as those from beef producers’ focus groups during the debriefing sessions.

**Figure 1:**
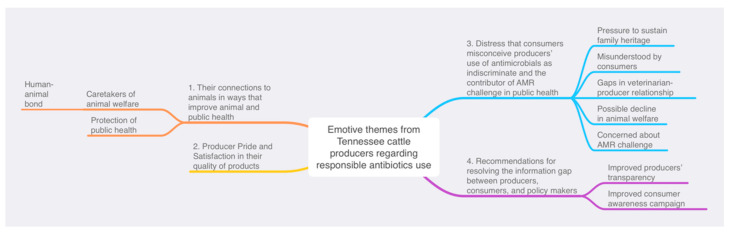
Regarding responsible use of antibiotics in cattle, Tennessee cattle producers emotively expressed the following four themes: (1) their connections to animals in ways that improve animal and public health; (2) pride in their quality of products; (3) their distress that consumers misconceive producers’ use of antimicrobials as indiscriminate and the contributor of AMR challenge in public health; and (4) recommendations for resolving the information gap between producers, consumers, and policy makers.

The producers emotively expressed the following four major themes: (1) their connections to animals in ways that improve animal and public health; (2) pride in their quality of products; (3) their distress that consumers misconceive producers’ use of antimicrobials as indiscriminate and the cause of AMR challenge in public health; and (4) recommendations for resolving the information gap between producers, consumers, and policy makers (Figure 1). One corresponding excerpt is provided for each of these themes. More selective excerpts supporting these themes are also available (see additional files 4–6).

### 3.1. Their Connections to Animals in Ways That Improve Animal and Public Health

Producers expressed a strong sense of connection with their animals, their families, and to their responsibility for the protection of public health. Producers emphasized their responsibility to prioritize animal welfare and health in their husbandry practices because doing so results in a higher profit, which helps producers care for their own families financially. They also emphasized that excellent public health practices impact their families as well. Thus, the two major sub themes identified as indicators of connections are (Section 3.1.1) caretakers of animal welfare: the human–animal bond, and (Section 3.1.2) protection of public health.

#### 3.1.1. Caretakers of Animal Welfare

Producers expressed moral responsibility to care for the animals in their farms. Rather than perceiving animals as mere commodities, some producers deemed care for the welfare of the animal as sacred, and many more reported humane husbandries as essential for financial solvency. Others expressed a similar feeling of closeness to their cattle as one might experience in caring for companion animals. Examples of these expressions include seeing and talking to these animals daily as well as describing them as big pets. These perspectives were expressed equally from both beef and dairy producers.

*If you’re a stockman, you have a care for that animal. It’s like a sacred trust that you’re supposed to take care of it. And it hurts you more than just your wallet when you lose one.*

*[No. 7, focus group 5].*

##### Human-Animal Bond

In the expression of humane treatments for animals, several human–animal comparisons, indicative of bonding connections, were made. These comparisons conveyed a strong emotional support a caretaker would give to an entrusted subject. Hence, producers expressed a sense of guilt when animal welfare fell short of their expectation.

*If your kids don’t get vaccinated, you took them to kindergarten, they’re going to get sick. … In my opinion, cattle ain’t no different. They’re our kids. God lets us take care of his creatures. We don’t go out here and just give shots. They cost money. We’re going to spend so much to take care of it and do the best that we can do. We want to treat them. We don’t want pinkeye…*

*[No. 8, focus group 1].*

#### 3.1.2. Protection of Public Health

Producers’ connection to their responsibility for the promotion of both animal and human health appears to influence their AMU practices. Protecting consumers from unwholesome products is a responsibility expressed by the producers. They believed that such protection starts with taking care of the animals and utilizing protocols that ensures that a few treated animals do not pose a public health risk in the food chain.

*And I totally agree with things that have been said here that we’re all in the business of raising healthy cattle to make a profit and have a positive bottom line. I can’t express how bad I would feel if I sold anything to anybody that cause them to be sick or worse. And I think the family farms in general and people producing meat animals share the same feeling.*

*[No. 3, focus group 4].*

### 3.2. Producer Pride and Satisfaction in Their Quality of Products

Notwithstanding the pressures of being caretakers and the increased expectations to produce a safer food, the producers took pride and expressed some gratification of their perceived role in society as well at the contributions they have made. Contrary to their current perceptions that regulations such as the VFD could threaten the survivability of their farming practices and a possibility of ending a family heritage, they spoke positively of other industry regulations that have shaped their products.

*And there has been a lot of good come out of regulations USDA and all that. That’s why we have the best product in the world is because of the regulations enforced to make a great product.*

*[No. 3, focus group 3].*

### 3.3. Their Distress That Consumers Misconceive Producers’ Use of Antimicrobials as Indiscriminate and the Contributor of AMR Challenge in Public Health

Producers expressed frustrations with activities surrounding successful execution of their practice. This distress was related to fears of going out of business and thus terminating a family heritage, feeling misunderstood by the consumers and frustrations in dealing with their veterinarians. Other reasons for distress include the possible compromise in animal welfare if AMU becomes highly restricted and the expectation that producers should play a vital role in curbing the AMR challenges that could develop in human medicine. Elaborations of these areas of concern and their excerpts are presented below.

#### 3.3.1. Pressure to Sustain Family Heritage

Part of producers AMU practices is hinged on the inward pressure to sustain a family heritage. Uncontrolled animal losses could put a producer out of business and with this comes a feeling of societal failure. Thus, some younger producers are under pressure to maintain a tradition by passing the inherited farming business to the next generation. This could mean doing all within their means to keep the animals healthy to the best of their abilities.

*The average age of the producers in the United States is 56-year-old. It’s a shrinking population. …Like I said, a hot week in August could wipe my operation, what my family worked for so many years [65 years] because of anaplasmosis. And that’s our biggest fear. It’s nothin’ else.*

*[No. 1, focus group 5].*

#### 3.3.2. Misunderstood by Consumers

Across the state, producers emotively expressed that consumers have the misconception that producers indiscriminately use antimicrobials. Such perceptions were attributed to misinformation and lack of public awareness about the day-to-day running of cattle farms and how they sometimes wake in the early hours of a cold morning to attend to sick animals. Some producers feel that policy makers and many consumers are out of touch with what happens in a typical cattle production system. Hence, consumer demands for antibiotic-free products impacts producers’ use of antimicrobials, leading to a reduction in or no usage of them.

*And I don’t want it to be against the law for me to keep that animal alive and healthy. I think there’s a terrible misconception because anybody that’s surviving today, you don’t survive a lifestyle because you’re spending all your money on antibiotics. You do everything management wise to prevent the need for it, whether it be sanitation, nutrition, daily removal of stress from the animal’s life—in your case, trying to keep out infectors from ’em. We do everything within our power management wise. And it’s a whole program, not just one step.*

*[No. 7, focus group 5].*

#### 3.3.3. Gaps in Veterinarian–Producer Relationship

The shortage of food animal veterinarians in certain areas of the state or the associated veterinarian consultation fees appear to stress producers and therefore affect their AMU practices. Invariably, some producers feel they are more knowledgeable about food animal care than some veterinarians. Amid this perceived knowledge, producers are frustrated because they are not permitted by law to write prescriptions for the treatment of their animals.

*…We work with animals on an everyday basis. So if we talk with a veterinarian that’s not used to dealing with food animals, quite frankly, we know a whole lot more about it than they do. But there’s really good food animal veterinarians that know way more than we do. But they’re the ones that are harder to find.*

*[No. 6, focus group 7].*

#### 3.3.4. Possible Decline in Animal Welfare

With a strong feeling of responsibility for caring for animal welfare, producers expressed a similar feeling of stress when the welfare of the animals is threatened due to AMU expectations or restrictions. Animals are often culled earlier than usual in order to avoid antibiotic treatments or to reduce potential revenue loss associated with adherence to withdrawal periods. Some producers hardly have a chance to treat mastitic cows because it was perceived not worth the time and money when she can be culled and replaced with a heifer. Such high rate of replacement of animals could be perceived as cruelty driven by either meeting consumer expectations or financial gains to the producer. This high turnover rate of cattle appears more common in dairy production systems.

*…I had an opportunity to meet a dairy farmer where he actually worked on an organic dairy farm in Oregon. And he called his farm labor—not allowed to use any antibiotics. He said you hear all these things about animal cruelty. He said you’ve never seen animal cruelty till you go on a farm that’s not allowed to use antibiotics. He said that’s the worse animal cruelty. He says you see a cow layin’ there for five days with one shot that would take care of it. And she lays there and suffers for five, ten days because the one shot that they can give her make everything okay. They feed her garlic and all these organic [inaudible]. He said that’s the worst animal cruelty you’ll ever experience.*

*[No. 4, focus group 5].*

#### 3.3.5. Concerned about Antimicrobial Resistance Challenge

Although some producers acknowledged that indiscriminate AMU could further the development of AMR genes in bacterial organisms and that it could pose a public health problem, they were reluctant in accepting that AMU in livestock has a major role in this potential public health challenge. Additionally, producers felt otherwise about the VFD regulation which was aimed at improving judicious AMU practices. Part of these ill feelings was that VFD had hindered a prevention tool that saved producers money. The VFD was perceived to increase the use of injectables and was hurtful to small producers who could not afford to judiciously execute the regulations as written currently.

*…Just to say that you would restrict to human use only, you would continue to see resistance increase—mother’s in a nursing home. In that environment, they culture in this patient. Well, she’s resistant now to everything but this. It has nothing to do with what she’s eating. The resistance is coming from the microorganisms that are becoming resistant to the drugs. And the humans are taking a lot more than the cattle are taking.*

*[No. 2, focus group 4].*

### 3.4. Recommendations for Resolving the Information Gap between Producers, Consumers, and Policy Makers

Producers were both convicted and victimized about some of the current practices that if strengthened could improve consumer confidence and create safer products. Their conviction arose from not fully getting their story out to the public. They felt victimized by food marketers taking advantage of public ignorance. By using buzzwords such as antibiotic-free labels on meat packages, these marketers shape consumer perceptions about AMU in cattle production. Because producers thought that policy makers and consumers are out of touch with what happens in a typical cattle production system, they felt that consumers need to be educated on how and why producers raise livestock the way they do.

#### 3.4.1. Improved Producers’ Transparency

Producers would like to be more open with information to the consumers as well as to their fellow producers about their AMU practices and how it relates to their animals and by-products. Communication of the right information is expected to better inform consumers and thereby boost confidence in their products.

*…And I don’t want to sound like devil’s advocate. As producers, we have to look at what we do from the perspective of the people on the other end of this chain. And we have to appreciate the fact that they are uninformed or misinformed sometimes. And we have to help them understand our objectives and goals and our priorities. And we’re going to have to become more transparent about what we do. We’re going to have to become more prepared to answer questions. And we’re going to have to be willing to discuss with them about our practices and what we do and why we do it the way we do it. If we’re able to articulate those things and help them understand that and we can erase the fear that they might get from propaganda or from people who have a different agenda than ours, if we can help them feel safe about the product that they’re eating and feel good about it, it won’t go as far as quickly as it will if the masses are scared and concerned and we’re close-mouthed about what we’re doing on our end because we feel defensive about it.*

*[No. 4, focus group 4].*

#### 3.4.2. Improved Consumer Awareness Campaign

Producers agreed that educating consumers is useful to solving the tension around AMU practices in livestock because consumers are believed to drive a lot of the regulation that is put on producers. Part of this education is informing the consumers about deceptive marketing practices aimed at appealing to the emotions of consumers about AMU practices and safety of their food. However, producers believe such claims are more about making additional money off the consumer and are not related to safety.

*…A lot of individuals, a lot of companies are taking advantage of that [consumer ignorance] by sticking that antibiotic-free sticker on there, jacking the price up. And both of them are antibiotic free because of withdrawal periods. But we’re taking advantage of the consumer…I think we have to educate consumers because ultimately, consumers drive a lot of the regulation that gets put on producer…*

*[No. 3, focus group 2].*

## 4. Discussion

The study presented here is a qualitative study designed to capture strong emotions of TN producers’ responsible use of antibiotics in cattle. The value of studies such as these is the opportunity to record strong emotions and determinative factors that are often missed in quantitative studies, providing useful tools for educators to design behavioral change campaigns that target those emotions. It is worth noting that these emotive themes or factors were solely the perceptions of the producers and therefore may not fully reflect all aspects of AMU practices encountered in typical beef and dairy operations, owing to the method of selection of participants and the number of dairy producers focus groups. With the purposive (non-random) selection participants and the few focus groups among dairy producers, other emotive views could have been missed among producers not enrolled in this study. Even among the beef producers’ group, the perceived data saturation was not absolute because more themes may have emerged, if additional focus groups were conducted [36]. Possible missed themes among dairy producer may be minimal, because dairy cattle production is not predominant in TN [35] and both focus groups yielded similar themes as those from beef producers’ focus groups. Nevertheless, the identified emotive themes were consistently expressed among each focus group and collectively provide vital insight into areas that should be considered in future targeted educational programs to producers aimed at altering AMU behaviors and improving judicious AMU practices in cattle production. These themes should not be seen as exhaustive rather a great starting point.

Consumers expects producers to look after their animals with diligence and care. No doubt caring for animals forms an important element of a farmers’ culture and personal identity [37] and producers perceive this as a crucial attribute of a good farmer [38]. Tennessee producers share similar sentiments but also expressed a recognition of a human–animal relationship. Strong connections between humans and animals, often referred to as the human–animal bond, has been well documented between humans and companion animals (particularly dogs and cats). These connections have been shown to protect both mental and physical ill health of humans by providing companionship and reducing the feeling of loneliness [39,40,41,42]. Specifically, the bond has been helpful among patients with autism, anxiety, and depression. Until the present study, such connections have been rarely reported between producers and their livestock, especially beef producers [43,44]. The human–animal bond has been perhaps more recognizable among dairy cattle producers compared to beef cattle producers based on the perception that dairy cattle have a closer, daily interaction with their human caretakers [45]. The concern about public health as observed in this study is contrary to that observed in other similar studies in the US [26]. Most farmers from South Carolina seemed unconcerned that AMU in animals could lead to resistance among farm workers [46] whereas 58% of conventional farmers from Ohio and Michigan disagreed that AMU in agriculture led to resistance bacterial infections in humans [47]. In the current study, where most participants were classified as beef cattle producers, participants recognized the importance of public health and emphasized the impact that it can have not only on their responsibility and kinship toward their cattle, but also on the health and wellbeing of their families and the public as well. Additionally, regardless of farm type, participants in each group demonstrated a perception that their animals are beings that should be treated with care. The possible reasons for the subtle differences in perceptions of relevance of AMU on public health among producers in various states in the US requires further exploration.

There is some sense of satisfaction that comes with knowing that one’s contributions are very important to the existence of another life. Proudly, producers derive satisfaction in their contributions toward providing a healthy protein source for the global population. Moreover, many producers expressed the belief that products of animal origin, such as cow’s milk, were healthier than manufactured products, such as soft drinks and plant-based drink products. In fact, these products were viewed as competition. A corresponding sentiment of the expanding world population and the growing need for high quality animal protein was expressed in concert with the feeling that participants would be directly involved in providing these food sources. These two factors could have influenced the pride exhibited by producers in the present study. Because the U.S. regulatory agencies for food perform constant regulatory oversight on the food products within their jurisdictions, the producers acknowledged such oversight to produce, arguably, the safest food in the world. However, concurrent with their satisfaction in their role is a constant challenge and stress of satisfying public expectations and demands.

In the last decade, the U.S. livestock industry has been increasingly faced with pressure to adjust practices in response to societal concerns, whether through governmental regulation or industry pressure through retailers responding to consumers. On one hand, government regulations such as the VFD have been perceived by small producers as an added burden that could end their business heritage [48]. On the other hand, consumers are primed that products raised without antimicrobials would reduce their chances of developing AMR challenges later in life [49]. Besides the potential public health risks, most consumers are supportive of complementary practices that improve animal welfare and because a marked reduction in animal welfare induced by intensive farming is often perceived to yield low quality products. In one study, consumers gained a more positive perception of a meat product and increased acceptability based on information about animal welfare and nutritional characteristics, however sensory properties played an important role in the determination of actual liking [50]. In other words, information on raising the animals in ways that optimize their welfare increased initial meat acceptability irrespective of how good the meat tasted. Inadvertently, these emotional expectations could easily translate to consumers’ zero tolerance for food products where AMU was evident in their production, because they may erroneously consider such production to be detrimental to animal welfare.

In reaction to consumer wants, several U.S. retailers and vendors (e.g., Walmart, Tyson foods, and McDonalds) have taken new positions on AMU and on farm animal welfare by demanding that suppliers engage in improved transparency measures and reporting standards regarding the treatment of animals [51]. Invariably, these demands are translated to producers, who must address the consumers expectations or risk going out of business. Undoubtedly, these expectations exert the distress experienced by producers. Compounding this distress is the shortage of food animal veterinarians in TN relative to other US states [52], with most shortage occurring in West TN counties [53] and the perception by some producers that they themselves know more about their animals than the veterinarians most readily available to them do. Cattle production in TN occurs predominantly in East and Middle TN and thus compounds the shortage and corresponding stressful veterinary relationships experienced by the producers in West TN. Recently, the U.S. Department of Agriculture initiated support programs to encourage retention of veterinarians in currently underserved communities throughout the country through the Veterinary Medicine Loan Repayment Program and the Veterinary Services Grant Program [54]. The perceived lack of knowledge about food animal medicine by producers is also currently being addressed for both practitioners and veterinary students alike, through targeted continuing education workshops [55] and increased exposure to immersive food animal experience in veterinary school [56].

Specifically, consumers form expectations of product quality attributes from both intrinsic (marbling and color of meat) and extrinsic cues (label, brand, origin, and production information) [57]. Consumers perceive as most important those production characteristics that relate to health and safety aspects rather than to animal welfare and environmental impact [58]. Often, consumers rely on product labels to decide which products meet their expectations and hence what product to purchase. However, these labels are often confusing and can be misleading. There may be usefulness in improving consumer awareness on detecting any possible deceptive labeling practices by vendor.

As acknowledged by the producers in this study, there are a few “bad eggs” among them, and they suggested the need for better transparency on their AMU practices. Old habits are often difficult to change, and this may be especially apparent for some producers who may not have been properly trained on the importance of judicious AMU in livestock. As suggested by previous studies [48,59,60,61], additional educational efforts of TN producers should focus on practical, cost-effective, and labor-effective alternatives to antimicrobial use (e.g., increased biosecurity, vaccination, and low-stress handling of livestock, and decreased stocking density) as a means of minimizing disease transmission. However, generic educational campaigns may not be so effective in changing long-formed habits. Embarking on an intensive effort to change peoples’ behavior requires an in-depth understanding of current behavior [22]. Because emotions are important aspects of behavior [29], several behavioral interventions have targeted people’s emotions as means of effecting change [30,31,32]. Specifically, psychological and behavioral determinants of farmers and veterinarians concerning AMU in food animal agriculture have been studied [24,25,26,27,28], because understanding these values can be a starting point to shaping future behavioral changes that could reduce the emergence of AMR challenge. Hence, targeted educational campaigns that focus on known emotions could lead to more successful behavioral changes on improved AMU practices.

## 5. Conclusions

In general, cattle producers perceived their use of antimicrobials to be judicious and feel that consumers should have confidence in the safety of animal products available at their local grocery stores. Producers felt they have a moral obligation to care for the animals entrusted to them as well as produce safe products because their families also consume these products. They felt victimized by food marketers’ deceptive product labels that take advantage of public ignorance. Nevertheless, producers were remorseful about their inadequacies in sharing their story. Thus, they suggested producers should be more transparent about their AMU practices and the public should have improved awareness to recognize deceptive product labels. Practically speaking, behavioral change is needed on both sides. Changing current behavior would be more successful if peoples’ emotions are targeted during educational campaigns. As a starting point, the present study has identified emotive views of producers regarding AMU in cattle. Prospectively, educators should incorporate these emotions in reshaping future communications.

## Data Availability

All data (focus group transcripts) pertaining to the manuscript cannot be shared in accordance with the confidentiality promise to the participants.

## Supplementary Materials

The following supporting information can be downloaded at: https://www.mdpi.com/article/10.3390/ani12162088/s1. Additional file 1: The first focus group interview guide; Additional file 2: The modified focus group interview guide; Additional file 3: Focus group participant characteristics; Additional file 4: Excerpts supporting Emotive themes from Tennessee cattle producers regarding responsible antibiotic use; Additional file 5: Consolidated criteria for reporting qualitative studies (COREQ): 32-item checklist; Additional file 6: Informed consent.
