# Supplementary material for: Emotive Themes from Tennessee Cattle Producers Regarding Responsible Antibiotic Use
Source: Animals (Basel). 2022 Aug 16;12(16):2088. doi: 10.3390/ani12162088 (PMC9405180; doi:10.3390/ani12162088)
Supplement: Supplementary file 1 [file animals-12-02088-s001.zip › animals-1815435-supplementary-Additional file 2.pdf]

The modified focus group interview guide

1. What kind of operation do you run?
2. How do you use antibiotics?
3. How does the veterinary feed directive affect your cattle production?
4. How easy is it to access a food animal veterinarian in your area?
5. Who or what influences your decision to start or discontinue the use of antibiotics? Please share things that are important to you when deciding to use antibiotics.
6. What is your opinion about restricting antibiotics for human use only? How would this affect your production practice? What do you believe about antibiotic resistance?
7. What can producers, consumers, veterinarians, and regulatory authorities do in order to make antibiotic use in cattle better?
8. What would you advice the secretary of health and human services to do about the causes and solutions of human and animal antibiotic resistance?
9. Please share other management practices or products besides antibiotics that you use to prevent or treat disease.
10. In your opinion, what specific type of information would you as cattle producers need and like to be receiving about antibiotic use? What is the best format?
11. What is important to you about this topic?
