# Supplementary material for: Emotive Themes from Tennessee Cattle Producers Regarding Responsible Antibiotic Use
Source: Animals (Basel). 2022 Aug 16;12(16):2088. doi: 10.3390/ani12162088 (PMC9405180; doi:10.3390/ani12162088)
Supplement: Supplementary file 1 [file animals-12-02088-s001.zip › animals-1815435-supplementary-Additional file 3.pdf]

Focus group participant characteristics (n = 62)

**Focus group**	**Cattle operation**	**Location**	**Herd size range**	**Gender of participants (total number)**
1	Beef	Johnson City, East Tennessee	40 - 80	All male (9)
2	Beef	Jefferson county, East Tennessee	20 - 200	All male (8)
3	Beef	McMinn county, East Tennessee	30 - 225	4 males, 1 female (5)
4	Beef	Dickson county, Middle Tennessee	40 - 135	All male (9)
5	Beef	McNairy county, West Tennessee	30 - 200	All male (8)
6	Dairy	Greene county, East Tennessee	50 - 1100	9 males, 2 females (11)
7	Dairy	Putnam county, Middle Tennessee	40 - 1000	11 males, 1 female (12)
