# Supplementary material for: Emotive Themes from Tennessee Cattle Producers Regarding Responsible Antibiotic Use
Source: Animals (Basel). 2022 Aug 16;12(16):2088. doi: 10.3390/ani12162088 (PMC9405180; doi:10.3390/ani12162088)
Supplement: Supplementary file 1 [file animals-12-02088-s001.zip › animals-1815435-supplementary-Additional file 4.pdf]

Consolidated criteria for reporting qualitative studies (COREQ): 32-item checklist

No	Item	Guide questions/description		Section where found
**Domain 1: Research team and reflexivity**				
**Personal characteristics**				
1	Interviewer/facilitator	Which author/s conducted the interview or focus group?	All the authors attended all the focus groups. The third author (EBS) moderated the focus group discussions.	Materials and Methods (Focus group design, structure, and procedure), paragraphs 1 &2.
2	Credentials	What were the researchers' credentials?	1st author (JEE): BVM, MVM, PgD, PhD Candidate<br>2nd author (MC): BS, DVM, PhD, DACVIM<br>3rd author (EBS): BA, MSSW, PhD<br>4th author (CCO): DVM, MS, PhD, DACVPM (Epi)	N/A
3	Occupation	What was their occupation at the time of study?	JEE: Graduate Research Assistant/PhD Candidate.<br>MC: Assistant Professor, Large Animal Clinical Sciences.<br>EBS: Director Veterinary Social work/ Clinical Associate Professor.<br>CCO: Assistant Professor, Epidemiology and Food safety.	N/A
4	Gender	Was the researcher male or female	Male: JEE, MC, CCO<br>Female: EBS	N/A

5	Experience and training	What experience did the researcher have?	JEE: Underwent qualitative research methods training while at graduate school and has experience in veterinary clinical practice, teaching senior veterinary students at a veterinary school.<br>CM: Has extensive experience in food animal veterinary practice.<br>EBS: Has wide experience in moderating group meetings.<br>CCO: Has wide experience in epidemiology and food safety.	N/A for JEE, MC and CCO.<br><br>Materials and Methods: (Research team and reflexity)
**Relationship with participants**				
6	Relationship established	Was a relationship established prior to study commencement	No relationship was established prior to study commencement.	N/A
7	Participant knowledge of the interviewer	What did the participants know about the researcher? e.g. personal goals, reasons for doing the research	Prior to the meetings, the participants knew nothing about the researchers. However, at the beginning of each focus group discussion, participants were informed about the purpose of the study as part of obtaining an informed consent prior to commencing with the discussions.	N/A
8	Interviewer characteristics	What characteristics were reported about the	Participants were informed that the moderator was a non-veterinarian with a	N/A

		interviewer/facilitator? E.g. bias, assumptions, reasons and interests in the research topic.	background in the behavioral sciences (social work).	
Domain 2: Study design				
Theoretical framework				
9	Methodological orientation and theory	What methodological orientation was stated to underpin the study? e.g. grounded theory, discourse analysis, ethnography, phenomenology, content analysis	Researchers were at liberty to use either inductive or the theoretical/deductive approach to thematic analysis.	Materials and Methods: data analysis section.
Participant selection				
10	Sampling	How were participants selected? E.g. purposive, convenience, consecutive, snowball	Participants were purposively selected.	Materials and Methods (Study design)
11	Method of approach	How were participants approached? E.g. face-to-face, telephone interview, mail, email	Participant recruitment e-mail was sent to the leadership of the Tennessee Cattlemen's Association (TCA) who then shared this email with TCA members and then purposively selected the volunteers for this study.	Materials and Methods (Study design)
12	Sample size	How many participants were in the study?	39	Materials and Methods (Study design)

13	Non-participation	How many people refused to participate or dropped out? Reasons?	No participant dropped out of the focus groups.	N/A
Setting				
14	Setting of data collection	Where was the data collected? Home, clinic, workplace?	Data was collected at county extension centers or at local restaurants were the focus groups were held.	Materials and Methods (Study design), paragraph 2.
15	Presence of non-participants	Was any one else present besides the participants and researchers?	No	N/A
16	Description of sample	What are the important characteristics of the sample? e.g. demographic data, date	Sex: 1 female and 38 male Perceived age: ranged from late twenties to early seventies. Reported herd size: 20 to 225 cattle	Results section: Focus group participant characteristics and summarized in the table 1 provided.
Data collection				
17	Interview guide	Were questions, prompts, guides provided by the authors? Was it pilot tested?	Yes, interview guide was provided. There was no specific separate pilot testing done. However, the interview guide was modified based on participant comments after the first focus group.	Materials and Methods (Study design), paragraph 2.<br><br>Additional files section: Additional files 1 and 2.
18	Repeat interviews	Were repeat interviews carried out? If yes, how many?	No repeat interviews were carried out.	N/A
19	Audio/video recording	Did the researchers use audio or visual	Data was video recorded.	Materials and Methods (Study design), paragraph 2.

		recording to collect the data?		
20	Field notes	Were field notes made during and/or after the interview or focus group?	Yes	Materials and Methods (Study design), paragraph 2.
21	Duration	What was the duration of the interviews or focus groups?	The focus groups lasted approximately 90 minutes.	Materials and Methods (Study design), paragraph 2.
22	Data saturation	Was data saturation discussed?	Yes. Data saturation was reached at the end of the 5th focus group.	Materials and Methods (Focus group design, structure, and procedure), paragraph 2.
23	Transcripts returned	Were transcripts returned to participants for comment and/ or correction?	No. Participants could not be identified since data was de-identified at collection for protection of human subjects in research.	N/A
Domain 3: Analysis and findings				
Data analysis				
	Number of data coders	How many data coders coded the data	All the four authors coded the data	Materials and Methods: data analysis section.
25	Description of the of the coding tree	Did authors provide a description of the coding tree?	The coding is described in the manuscript	Materials and Methods: data analysis section.
26	Derivation of themes	Were themes identified in advance or derived from the data?	Themes were not identified in advance. Final themes presented in the manuscript were arrived at after two review & harmonization meetings to compare individual data coding.	Materials and Methods: data analysis section.

27	Software	What software, if applicable, was used to manage the data?	NVivo qualitative data analysis Software; QSR International Pty Ltd. Version 11, 2017 was used.	Materials and Methods: data analysis section.
28	Participant checking	Did participants provide feedback on the findings?	No. Participants were de-identified, hence could not be traced back.	N/A
Reporting				
29	Quotations presented	Were participant quotations presented to illustrate the themes/findings? Was each quotation identified e.g. participant number	Yes, quotations were presented verbatim (in participants' own words) to illustrate the themes/findings. Each quotation was identified by participant number, except for a few un-identified participants.	Results section
30	Data and findings consistent	Was there consistency between the data presented and the findings?	Yes	N/A
31	Clarity of major themes	Were major themes clearly presented in the findings?	Yes	Results section and summarized in the thematic map.
32	Clarity of minor themes	Is there a description of diverse cases or discussion of minor themes?	Yes	Results section and summarized in the thematic map.
