# Supplementary material for: Emotive Themes from Tennessee Cattle Producers Regarding Responsible Antibiotic Use
Source: Animals (Basel). 2022 Aug 16;12(16):2088. doi: 10.3390/ani12162088 (PMC9405180; doi:10.3390/ani12162088)
Supplement: Supplementary file 1 [file animals-12-02088-s001.zip › animals-1815435-supplementary-Additional file 5.pdf]

Excerpts supporting Emotive themes from Tennessee cattle producers regarding responsible antibiotic use

3. Results

3.1. Their connections to animals in ways that improve animal and public health

3.1. A. Caretakers of Animal Welfare

If you're a stockman, you have a care for that animal. It's like a sacred trust that you're supposed to take care of it. And it hurts you more than just your wallet when you lose one. [No. 7, focus group 5].

…These cows are our livelihood. And we have to go to sleep at night knowing how we take care of these cows. And for me to go to sleep at night knowing I've let a bunch of cows go with one eye because I didn't want to give them only $200.00, that's just not something I can do. [No. 6, focus group 7].

I want my animals to have the best. With my pig operation, sir, I tell folks that the temperature we try to control it better than I control the temperature in my personal house. If that animal is healthy, they're going to produce more. You asked about the quality of the life. Well, as a producer, I want their quality of life to be the best. And whether it's their health or whether it's the amount of food that's in front of them – you know, if we've got good, lush pastures for our cattle, then they have the opportunity to be healthier and happier. [No. 4, focus group 3].

We've also used antibiotics heavily on old bulls. It takes a large bull. His feet are bad and should've already sold him. He's like a big pet right now…a 2500-pound pet. And we're trying to save those feet. We've been doctoring him long-term under the vet's care, too. And it appears to be successful. [No. 7, focus group 4].

I think I've used some feed a couple times. I don't like to use medicine – I only do it here in place of that because I just think it's morally wrong. I don't use anything unless I have to. But as small as I am, I see 'em every day. And I talk to 'em every day, ya know, [inaudible]. [No. 3, focus group 5].

3.1. Ai: Human-Animal Bond

If you had a kid that was sick, right or wrong, you'd do whatever you could to get the job done. [No. 4, focus group 5].

… like No. 11 said. I raise three kids. And I've got two grandkids. If I heard they were sick, I'm going to not do anything about it – I think we need to retrain that ability to treat a sick animal. [No. 8, focus group 7].

… If your kids don't get vaccinated, you took them to kindergarten, they're going to get sick. …In my opinion, cattle ain't no different. They're our kids. God lets us take care of his creatures. We don't go out here and just give shots. They cost money. We're going to spend so much to take care of it and do the best that we can do. We want to treat them. We don't want pinkeye….[No. 8, focus group 1].

3.1. B. Protection of Public Health

And I totally agree with things that have been said here that we're all in the business of raising healthy cattle to make a profit and have a positive bottom line. I can't express how bad I would feel if I sold anything to anybody that cause them to be sick or worse. And I think the family farms in general and people producing meat animals share the same feeling. [No. 3, focus group 4].

If you eliminate the antibiotics where we can't use them or have harder access to them like that, you're going to see sicker cattle go into the food chain. Our costs are going to go up because we're going to lose more. And you're going to see sicker animals go into the food chain – with no telling what – that could be avoided. [No. 7, focus group 4].

…Anytime we do any antibiotics, whether it be injectable or anything else, we write it down. We track the withdrawal times. And if we do take something to sell, which we don't sell many at the sale now – but if we take some to sell, we'll look back and make sure nothing's had a shot or anything within a timeframe that's going too early. Probably everybody here that says it's not hard to do. It's just the right thing to do. [No. 7, focus group 3].

I want the consumers to have a good product because we feed this to our families, too. We're not gonna do somethin' to jeopardize the people that we sell the product to because we need them to make business. [No. 1, focus group 5].

I think this antibiotic topic we've got fake news. We all hear about stuff like that. A lot of things are misrepresented in the news media. You can take something small and blow it up to nothing. I'm allergic to penicillin. And enough of it in milk will really do me in. And I can only imagine a small child. So, this is important to all of us whether we want to believe it or not. We've got to keep a clean product in order to have a good name and sell it. This is a very important topic. [No. 9, focus group 6].

I'll use some marketing terms or some mixed markets. But there's nothing wrong with all organic. There's nothing wrong with all natural. There's nothing wrong with properly fed antibiotic feeds if you can sell it and if you can afford to buy it. They say that we have a rising food cost in America or around the world. There's a greater need. When I came into this field, a farmer produced enough, I think, to feed himself and 46 other people. The last number I saw was 168. I don't know where it's at today. But if we have hungry people, can they afford to buy or pay for what it costs to produce organic milk? There's not a thing in the world wrong with it. I've had some. If it was true organic milk. All I got to do is find what's on the label. Did it hurt the pocketbook? Well, sure we did. Are we willing to pay for it? [No. 4, focus group 3].

3.2. Producer Pride and Satisfaction in their quality of products

And there has been a lot of good come out of regulations USDA and all that. That's why we have the best product in the world is because of the regulations enforced to make a great product. [No. 3, focus group 3].

… we have so much competition from other drinks out there. And the more that we keep our product healthy – I mean, we have the best product in the world. And we're having to compete against Coca-Cola, which is probably one of the worst products in the world. But we've got to have such a good image to promote our product that the cleaner, the better we are, the more perfect we are, the better it benefits us. [No. 3, focus group 6].

… When you're dealing with the food chain, a country that cannot feed itself will fall. …I've said 2050, ten million people, we've got to feed them… We're dealing with a society that the consumers have a good product, an improvement product, the safest product in the world. [No. 1, focus group 5].

I would say that on the same thing he's talking about. The somatic cell level is so low now that there ain't a dairy farmer in this room that ain't had a good operator because the economics has done gotten rid of the guys that couldn't make it. They still won't give some in these economic conditions. There's no need the public being in outcry over us because what's left the best. [No. 11, focus group 7].

3.3. Their distress that consumers misconceive producers' use of antimicrobials as indiscriminate and the contributor of AMR challenge in public health

3.3. A. Pressure to Sustain Family Heritage

The average age of the producers in the United States is 56-year-old. It's a shrinking population. … Like I said, a hot week in August could wipe my operation, what my family worked for so many years [65 years] because of anaplasmosis. And that's our biggest fear. It's nothin' else. [No. 1, focus group 5].

… If you've been feedin' 'em what they need, most of the time, you'll be more healthy. You just have to do what ya have to do. Money ain't the most important thing. I thought of my family. My family been havin' 'em for years. My own pet – when I call 'em to come, they come. They know I'm gonna take care of 'em and do my best. It ain't all about money. It's all about bein' with 'em, enjoyin' what ya do. [No. 6, focus group 5].

You learn from financial loss. But your heart never forgets them losses. And you learn from that entire – that-a-boys and them daddy boys make you what you are. I'm very passionate about this because this is my family's work. And I did it to make my family's work – the legacy that will live past me. [No. 1, focus group 5].

3.3. B. Misunderstood by the Consumers

There's a difference in a calf that's been on good feed versus a calf been on grass. I can tell you that. I guarantee you, 90 percent of the people that say we're going to quit doing antibiotics has never fed a calf in their life. They've never honestly laid hands on a calf. They've never doctored a calf. But when they go to the store, they want a prime piece of meat to eat. But they never had any idea of what goes to get that piece of meat that's on the plate [Unidentified speaker, focus group 3].

… And I didn't know it when they come there that they were interested in organic. And this lady was there. The organic milk, you're not going to give the vaccines and stuff that we need. Well, at that point, I was under the impression – but I asked her did you give your children vaccines and the medicines they need to get well? Well, of course. I said would you give your children milk from animals that were not treated the same way? And she still had her mouth open when she left. You feed your children a product that has not been handled the same way you would handle your children. [No. 1, focus group 7].

...It's no fun to go get them up and doctor them. I mean, you just don't do it to have something to do. Most of us would rather just sit in a chair and watch them grow. That's the thing about it. That's a lot a work to go out there. And you got to understand, too, when you're putting this medicine in them, whether it's in the feet or injectable, it's costing you money. I mean, a bottle of Draxxin's $1,800.00. [No. 2, focus group 2].

And I don't want it to be against the law for me to keep that animal alive and healthy. I think there's a terrible misconception because anybody that's surviving today, you don't survive a lifestyle because you're spending all your money on antibiotics. You do everything management wise to prevent the need for it, whether it be sanitation, nutrition, daily removal of stress from the animal's life – in your case, trying to keep out infectors from 'em. We do everything within our power management wise. And it's a whole program, not just one step. [No. 7, focus group 5].

If I put an animal in a fence, I feel morally responsible to try to do the best possible job I can do. We're not here to try and create problems. And we're not here to get a bunch a money that we don't need to spend. We wanna keep our animals healthy. But the lobbyists for [inaudible] have got more money and more presence than we do, and that's what's drivin' all this – tryin' to kill us off so this won't be part of the food chain. They don't understand that. They go by and see all the big feed lot, and they think everybody's that way. I think it's misinformation that's been delivered that they put out to try to paint the attitudes toward us. [No. 3, focus group 5].

Them people do not know the mornings that you're up at 3:00 in the morning trying to get a cold, wet calf that's half iced over to nurse a cow. They do not know that struggle. This is not somethin' that's took on by the faint of heart. [No. 1, focus group 5].

3.3. C. Gaps in Veterinarian-Producer Relationship

…We work with animals on an everyday basis. So if we talk with a veterinarian that's not used to dealing with food animals, quite frankly, we know a whole lot more about it than they do. But there's really good food animal veterinarians that know way more than we do. But they're the ones that are harder to find. [No. 6, focus group 7].

I can evaluate a problem. I can't write you a script to get medicine to fix it. So we're still stuck. [No. 7, focus group 5].

We have access to a group of veterinarians about an hour, 45 minutes away. They deal mainly with beef cattle on the large animal side. They know very little about dairy produce restrictions and that sort of thing. Like somebody said earlier, they ask me what we should use? When you get the bill, it kind of hurts your feelings. [No. 13, focus group 7].

3.3. D. Possible Decline in Animal Welfare

…Milk companies and everybody wants us to have quality milk. And they want us to have good animal husbandry. And they want us to have good animal welfare. The biggest welfare problem I see is turning cows over so fast. These cows won't live to be very old. I don't like it a bit. But you've got to turn them. [No. 11, focus group 7].

…I had an opportunity to meet a dairy farmer where he actually worked on an organic dairy farm in Oregon. And he called his farm labor – not allowed to use any antibiotics. He said you hear all these things about animal cruelty. He said you've never seen animal cruelty till you go on a farm that's not allowed to use antibiotics. He said

that's the worse animal cruelty. He says you see a cow layin' there for five days with one shot that would take care of it. And she lays there and suffers for five, ten days because the one shot that they can give her make everything okay. They feed her garlic and all these organic [inaudible]. He said that's the worst animal cruelty you'll ever experience. [No. 4, focus group 5].

A couple years ago, I thought I'd possibly transition into organic. And I had a pinkeye outbreak in my calves. I talked to several organic producers about what they would do to handle that pinkeye outbreak. What we just need is one-eyed cows. I can clear them up. These cows are our livelihood. And we have to go to sleep at night knowing how we take care of these cows. And for me to go to sleep at night knowing I've let a bunch of cows go with one eye because I didn't want to give them only $200.00, that's just not something I can do. [No. 6, focus group 7].

3.3. E. Concerned about Antimicrobial Resistance Challenge

Prior to January 1, yes, I kept antibiotics in mineral because two years ago, we had an outbreak of anaplasmosis...Now thanks to this, my understanding of it is I have to have an outbreak. I can't permit. So I've gotta sit here on pins and needles and hope that I'm not gonna lose fetuses and the little cows. And you guys being veterinarians you know how hard to diagnose anaplasmosis is. You might draw a blood sample and send it to a lab. By the time it gets there, there's another conclusive event. It takes a large animal vet onsite. And we don't have one of those. We have a very useful tool that I saw no harm to humans because anaplasmosis does not cross species. I didn't see that being a danger to humans, causing any resistance or anything. But now I've gotta wait for a loaded gun to shoot me before I can do anything. [No. 7, focus group 5].

…Just to say that you would restrict to human use only, you would continue to see resistance increase – mother's in a nursing home. In that environment, they culture in this patient. Well, she's resistant now to everything but this. It has nothing to do with what she's eating. The resistance is coming from the microorganisms that are becoming resistant to the drugs. And the humans are taking a lot more than the cattle are taking. [No. 2, focus group 4].

3.4. Recommendations for resolving the information gap between producers, consumers, and policy makers

3.4. A. Improved Producers' Transparency

…And I don't want to sound like devil's advocate. As producers, we have to look at what we do from the perspective of the people on the other end of this chain. And we have to appreciate the fact that they are uninformed or misinformed sometimes. And we have to help them understand our objectives and goals and our priorities. And we're going to have to become more transparent about what we do. We're going to have to become more prepared to answer questions. And we're going to have to be willing to discuss with them about our practices and what we do and why we do it the way we do it. If we're able to articulate those things and help them understand that and we can erase the fear that they might get from propaganda or from people who have a different agenda than ours, if we can help them feel safe about the product that they're eating and feel good about it, it won't go as far as quickly as it will if the masses are scared and concerned and we're close-mouthed about what we're doing on our end because we feel defensive about it. [No. 4, focus group 4].

When you're running around doctoring, you're not making no money. And instead of that calf being gone in 120 days, you might have him 200-250 days. If you're going to keep a reputation in this business, one that you've doctored like that, you're not going to send him to a feed yard because more than likely, his lungs are going to be

damaged. And you're going to take him back to the stock yard and put him off on your friend and just not say nothing. That's the truth of the matter. You've got to keep your reputation if you're going to sell cattle and get anything out of it. You don't want to send a load of 800-pound steers to a feed yard and have 8 or 10 of them that you know you've treated more than twice. I mean, you can get along treating them pretty good two times. After that, you just well quit 'cause your medicine cost – they don't respond the first two times, it's over. Or that's my take on it. [No. 2, focus group 1].

I think that communication is the key and bein' open-minded to each other, workin' together – people knowin' everything [inaudible]. Human education is great. [inaudible] – be open-minded to each other. Sometimes we just don't work with each other. We've gotta mass it one way – and this has to be – another guy says this has to be – in addition to what somebody has to see and try and understand where they comin' from. I think understandin' would be the key to it all. I think there ain't no problem we can't work out. [No. 6, focus group 5].

… if a guy's got six cattle and he's doctored one and it's not getting better, you know where it's going. He aingt worried about no withdrawal. He's taking that cow to the stock market. That's the problem. [No. 3, focus group 3].

We follow all the beef guidelines and what you're supposed to do with withdrawal periods…. Bad eggs – I understand. There's gonna be people that are gonna do things to circumvent law that's not right. [No. , focus group 5].

Back to maybe in addition to information, we have to reemphasize and make sure that the information out there is correct information based on what we know to be true rather than some of the propaganda that's misleading. And sometimes it's downright incorrect. I think so many of us – and speaking personally for myself, it's a lot easier just to mind your own business and stay home and take care of what needs taking care of than it is to get involved with situations like this and try to make a difference. [No. 3, focus group 4].

This point may be kind out in left field. But then again, it might not be as far out in left field as some might think. Lots of cattle meetings that I attend and agriculture sponsored to the meat industry, I be there's some mind-set to the fact that HSUS are on a mission to put an end to animal agriculture as we know it. And misinformation and certain things that have been talked about tonight, I think will possibly lend strength to their goal. And we've got to do whatever we can to counteract that and see that the right information gets before the public. [No. 3, focus group 4].

3.4. B. Improved Consumer Awareness Campaign

… A lot of individuals, a lot of companies are taking advantage of that [consumer ignorance] by sticking that antibiotic-free sticker on there, jacking the price up. And both of them are antibiotic free because of withdrawal periods. But we're taking advantage of the consumer… I think we have to educate consumers because ultimately, consumers drive a lot of the regulation that gets put on producer… [No. 3, focus group 2].

I think he's right. A lot of times people assuming things aren't true just by word of mouth. I had a customer the other day talking about somebody coming to visit his dairy and never seen cows being milked and walked in there and just amazed at it and talking about whether he's given antibiotics to his cattle and weren't they using hormones in there and all this stuff. He explained them the truth of it. There were no hormones being used in his milk, no antibiotics going in there. They had heard everything on social media or the television or whatever and believed

everything they heard. So yes, I think education of the public that have no idea how animals are produced and how their food is produced would be a good idea. [No. 5, focus group 3].

We've got big messes with these want to be milk drinks, too. That needs to be knocked plum off the counter. That shouldn't even exist. I don't know how it ever got through. But that should never be. There's only one milk. You can look up the definition of milk. And it says from a mammal. And I don't believe there's a mammal in a tree. They get a check every week. That tree ain't breathing. [No. 6, focus group 6].

I think part of the problem with the public is our milk marketing. This jug of milk says antibiotic and hormone free and this one does not. So they assume that that one has antibiotics in it, which falls into antibiotics in milk and all this antibiotic resistance and stuff like that when no milk has antibiotics in it. But they just don't know that. They're just not educated. [No. 12, focus group 7].
