# Supplementary material for: Emotive Themes from Tennessee Cattle Producers Regarding Responsible Antibiotic Use
Source: Animals (Basel). 2022 Aug 16;12(16):2088. doi: 10.3390/ani12162088 (PMC9405180; doi:10.3390/ani12162088)
Supplement: Supplementary file 1 [file animals-12-02088-s001.zip › animals-1815435-supplementary-Additional file 6.pdf]

College of Veterinary Medicine
Biomedical & Diagnostic Sciences
2407 River Drive – Room A201
Knoxville, TN 37996-4542
main: 865-974-5571
fax: 865-974-5616
vetmed.tennessee.edu

Informed Consent Form

Drivers of antimicrobial use practices among cattle producers in Tennessee: a qualitative study

This informed consent form is for cattle producers in Tennessee who we are inviting to participate in our research entitled "Drivers of antimicrobial use practices among cattle producers in Tennessee: a qualitative study".

Name of Principal Investigator: John Eddie Ekakoro.

Name of Co-Principal Investigator: Chika C. Okafor.

Name of Co-Investigator: Marc Caldwell.

Name of Co-Investigator: Elizabeth B. Strand.

This informed consent form has two parts:

- Information sheet (to share information about the study with you).
- Certificate of consent (for signatures if you choose to participate).

You will be given a copy of the full informed consent form.

Part one: Information sheet

Introduction

I am **John Eddie Ekakoro**, a PhD student in the Comparative and Experimental Medicine program at the University of Tennessee, Knoxville. I am conducting research to determine factors influencing antimicrobial use practices in cattle production in Tennessee as part of my PhD. With your participation in our invited focus groups, we will be providing information about some of the public concerns for the use of antimicrobials and inviting you to participate in this research. If you would like more information than what is provided in this document you can contact any of the researchers to ask questions about the research. The researchers' contact information is included below.

Purpose of the research

Antimicrobial medicines play a very important role in livestock production, including the treatment or prevention of infections. In some cases, microbes (germs) develop resistance to certain antimicrobial agents. Diseases caused by these microbes are

IRB NUMBER: UTK IRB-17-03702-XP
IRB APPROVAL DATE: 05/03/2017
IRB EXPIRATION DATE: 05/02/2018

continuing to challenge farmers and veterinarians, leading to increased cattle production costs and economic losses in farms and causing difficult-to-treat illnesses in people. Indiscriminate use of antimicrobials in animals and humans is perceived by the public as an important factor contributing to the development of antimicrobial resistance. But, antimicrobial resistance challenge has been equally observed in naïve animals without evidence of previous exposure to antimicrobials. Optimal use of antimicrobial agents is critical for a healthy and profitable livestock production. It is therefore important to identify current practices around antimicrobial use in Tennessee livestock.

Through this study, we anticipate obtaining insights into your perceptions, opinions, concerns, and experiences about antimicrobial use practices in cattle production. Importantly, your responses will clarify the objectives of a follow-up questionnaire that will be distributed to Tennessee cattle producers at large. Overall, lessons learned from your participation will help to advance the implementation of best practices towards antimicrobial use in cattle production.

Type of research intervention

This research will involve your participation in a focus group discussion that will take about one and a half hours (90 minutes). The focus group will comprise approximately 10 participants. Each session will be audio and video recorded and transcribed for later analysis.

Participant selection

You are being invited to take part in this research because we feel that your experience as a cattle producer will contribute much to our understanding and knowledge of antimicrobial use practices in cattle production in Tennessee.

Voluntary participation

Your participation in this study is entirely voluntary. It is your choice whether to participate or not. Either choice will not influence your relationship with the researchers or the University of Tennessee. You may stop your participation at any time.

Procedures

In this meeting would be other 7-9 producers, yourself, and the researchers from the University of Tennessee. The discussion will be guided (moderated) by Dr. Elizabeth B. Strand while Dr. Marc Caldwell will introduce the research rationale and goals. You would have time to answer any questions about the research. Next, we will ask a series

IRB NUMBER: UTK IRB-17-03702-XP
IRB APPROVAL DATE: 05/03/2017
IRB EXPIRATION DATE: 05/02/2018

of questions about antimicrobial use practices. After each question there will be time for discussion in which we desire to listen to your knowledge and experience on each subject. The moderator will create a group discussion where everyone's opinion is heard and considered.

The questions will be clustered under the following topic headings: current antimicrobial use, factors influencing the decision to use antimicrobials by cattle producers in Tennessee, perceptions of antimicrobials and their usage, and future options.

The intent of these questions is to prompt your opinions about antimicrobial use practices. We will also talk about your awareness, and opinion on antimicrobial use practices of cattle producers across the industry to gain a different perspective.

The focus group discussion will take place in (venue would be inserted here). The entire discussion will be audio and video recorded. Excerpts of the audio/video may be used for teaching purposes at the University of Tennessee. All voice recordings shall be anonymized. Participant voices in audio recordings shall be tweaked and participants' faces in video recordings shall be blurred, if the audio/video recording is to be used for teaching purposes. Tweaking of voices will make participant voices unidentifiable to listeners. Blurring of video recordings will make participant faces unidentifiable to viewers. The recording of this discussion will be kept in the Department of Biomedical and Diagnostic Sciences at the University of Tennessee, College of Veterinary Medicine on a password-protected computer in room A326 VMC. Your responses will be transcribed and your name or other identifying information will not be included in the transcription. The transcribed information will be used to address the research goals for this project (see 'Purpose of Research").

Duration

The group discussion will be held once and should take about one and a half hours (90 minutes).

Benefits

There will be no direct benefit to you, but your participation is likely to help us find out more about how to develop targeted approaches to improve best use practices for antimicrobials in cattle. These targeted approaches could be adopted in other states thereby leading to the realization of prudent use of antimicrobials in cattle production in the United States.

IRB NUMBER: UTK IRB-17-03702-XP
IRB APPROVAL DATE: 05/03/2017
IRB EXPIRATION DATE: 05/02/2018

Reimbursements

You will not be provided with any incentive to take part in this research. However, one catered meal (approximately $12 meal) will be provided to you before the start of the focus group session. You would be required to specify any dietary restrictions prior to the focus group meeting.

Confidentiality

We shall ask you and others in the group not to talk to people outside the group about what was said in the group. We will, in other words, ask each one of you to keep what was said in the group confidential. You should know, however, that we cannot stop or prevent participants who were in the group from sharing things that should be confidential.

The information that we collect from this research project will be kept private and any identifiable information about you will be anonymized during manuscript reporting. Only the researchers will know what your number is and we will keep that list on a password-protected computer in room A326 VMC of the UTCVM. This list will be separated from transcribed records.

Sharing the results

The results of this research will be published in a scientific journal that will be accessible to the public. Published findings will not identify you in any way.

Right to refuse or withdraw

You do not have to take part in this research if you do not wish to do so and choosing to participate will not affect you in any way. You may choose not to answer any question you find uncomfortable answering or you may stop participating in the discussion at any time that you wish. You do not have to share any knowledge that you feel uncomfortable sharing.

Risks

Possible risks to participants would be minimal and would not be greater, in and of themselves, than those ordinarily encountered in daily life. For questions or concerns, the primary investigators' information is available to you.

Who to contact

If you have any questions, you may contact any of the following:

IRB NUMBER: UTK IRB-17-03702-XP
IRB APPROVAL DATE: 05/03/2017
IRB EXPIRATION DATE: 05/02/2018

1. **John Eddie Ekakoro, BVM, MVM, PGDip., PhD student.**

   Department of Biomedical and Diagnostic Sciences

   Comparative and Experimental Medicine graduate program

   College of Veterinary Medicine

   University of Tennessee

   2407 River Drive-Room A201

   Knoxville, TN 37996-4542

2. **Chika C. Okafor, DVM, MS, Ph.D., DACVPM (Epi).**
   Department of Biomedical and Diagnostic Sciences College
   of Veterinary Medicine
   The University of Tennessee
   2407 River Drive-Room A201
   Knoxville, TN 37996-4542
   Telephone: 865-724-5223
   Email: okaforch@utk.edu

3. **Dr. Marc Caldwell, BS, DVM, Ph.D., DACVIM.**
   Department of Large Animal Clinical Sciences,
   College of Veterinary Medicine,
   The University of Tennessee,
   2407 River Drive,
   Knoxville, TN 37996-4542

4. **Dr. Elizabeth B. Strand, BA, MS, Ph.D.**
   Department of Biomedical and Diagnostic Sciences College
   of Veterinary Medicine
   The University of Tennessee
   2407 River Drive-Room A201

IRB NUMBER: UTK IRB-17-03702-XP
IRB APPROVAL DATE: 05/03/2017
IRB EXPIRATION DATE: 05/02/2018

Knoxville, TN 37996-4542

This research has been reviewed and approved by The University of Tennessee Institutional Review Board, which is a committee whose task it is to make sure that research participants are protected from harm. If you have any pertinent questions or concerns about your rights as a participant, contact the University of Tennessee Institutional Review Board compliance officer at 865-974-7697 or utkirb@utk.edu.

Part two: Certificate of consent.

I have been invited to participate in research about drivers of antimicrobial use practices in cattle production in Tennessee.

I have read the foregoing information, or it has been read to me. I have had the opportunity to ask questions about it and any questions I have been asked have been answered to my satisfaction. I consent voluntarily to be part of this study.

Name of Participant………………………………………………………………………………

Signature of Participant…………………………………………………………………..

Date………………………………………………………………………………………

Month/day/year

Consent to the audio/video recording and use of excerpts for teaching purposes

I consent voluntarily to the audio/video recording of the focus group discussion. Excerpts of the audio/video may be used for teaching purposes at the University of Tennessee.

Name of Participant……………………………………………………………

Signature of Participant…………………………………………………………………

Date……………………………………………………………………………………

Month/day/year

IRB NUMBER: UTK IRB-17-03702-XP
IRB APPROVAL DATE: 05/03/2017
IRB EXPIRATION DATE: 05/02/2018
